# SMC4, which is essentially involved in lung development, is associated with lung adenocarcinoma progression

**DOI:** 10.1038/srep34508

**Published:** 2016-09-30

**Authors:** Chengli Zhang, Manchao Kuang, Meng Li, Lin Feng, Kaitai Zhang, Shujun Cheng

**Affiliations:** 1 State Key Laboratory of Molecular Oncology, Department of Etiology and Carcinogenesis, National Cancer Center/Cancer Hospital, Chinese Academy of Medical Sciences and Peking Union Medical College, Beijing, 100021, China

## Abstract

Structural maintenance of chromosome 4 (SMC4) is a core subunit of condensin complexes that mainly contributes to chromosome condensation and segregation. Our previous study demonstrated that the gene expression profile during lung development is of great values for the study of lung cancer. In this study, we identified SMC4 through co-expression network analysis and clique percolation clustering using genes that constant changes during four stages of lung development. Gene ontology and KEGG pathway enrichment analysis demonstrated that SMC4 is closely related to cell cycle, cell adhesion, and RNA processing in lung development and carcinogenesis. Moreover, SMC4 is overexpressed in lung adenocarcinoma tissues and acts as an independent prognostic factor. SMC4 knockdown significantly inhibits the proliferation and invasion of A549 cells. Furthermore, we found that SMC4 interacts with DDX46 (DEAD-box helicase 46). In conclusion, the pivotal role of SMC4 in lung development and carcinogenesis suggests that genes with a similar expression pattern to SMC4 in lung development may also contribute to lung cancer progression. The identification of genes that are essentially involved in development through a comparative study between development and cancer may be a practical strategy for discovering potential biomarkers and illuminating the mechanisms of carcinogenesis.

Lung cancer is the leading cause of cancer death worldwide, and lung adenocarcinoma (ADC) accounts for almost half of all lung cancer cases^1^,^2^. Due to tumor heterogeneity, lung ADC patients at similar clinical stages may exhibit marked inter-individual variations in tumor progression and response to therapy, leading to substantially different clinical outcomes^3^,^4^,^5^,^6^. Therefore, it is necessary to explore the mechanism underlying lung ADC tumorigenesis and evaluate the diversity in molecular expression profiles among lung ADC patients, and the findings from these studies may eventually help clinicians deliver precise therapy and improve the survival rate of lung ADC patients.

Mounting lines of evidence suggest a close relationship between tumorigenesis and developmental processes^7^. Several studies have demonstrated that cancer cells share many similarities with embryonic cells. First, cancerous tissues are generally undifferentiated, and some tumor types even exhibit embryonic tissue organization. Second, increased cell mobility during tumor invasion and metastasis imitates the migratory behavior observed during development. Third, the infinite proliferation capability of tumor cells is consistent with the characteristics of pluripotent stem cells or totipotent stem cells during the embryonic stage. Fourth, the abnormal re-activation of developmental genes in tumor cells is common. Fifth, shared signal transduction pathways (e.g., Wnt, Hedgehog, and Notch) have been clearly elucidated in both cancer cells and embryonic cells^8^,^9^. In addition, several studies have indicated that cancer cells mimic the gene expression patterns exhibited by the corresponding organ during the early developmental stages^10^,^11^,^12^,^13^,^14^.

Condensin complexes are heteropentamers composed of two structural maintenance of chromosome (SMC) subunits (SMC2 and SMC4) and three non-SMC subunits. The structure and function of SMC4 are evolutionarily conserved from bacteria to humans. SMC4 belongs to the SMC family of chromosomal ATPases, which have two highly conserved nucleotide-binding Walker A and B motifs on the N- and C-terminal domains and a moderately conserved hinge motif on the central domain^15^,^16^,^17^. The most widely reported function of SMC4, as well as the remaining subunits of whole condensin complexes, is chromosome condensation and segregation. Accumulating lines of evidence demonstrate that SMC4 may participate in a wide variety of nonmitotic chromosome functions, such as maintenance of the silenced state of gene expression, heterochromatin organization, and DNA repair^18^. Recent studies showed that SMC4 is involved in liver and colon tumorigenesis^19^,^20^,^21^,^22^. Although a few studies have focused on the relationship between SMC4 and tumorigenesis, the underlying molecular mechanisms need to be further explored. Additionally, the role of SMC4 in lung ADC carcinogenesis has not yet been reported.

In our study, we identified SMC4 through the construction of a co-expression network of genes that exhibit constant changes in expression during lung development. Furthermore, we found that SMC4 is upregulated in lung ADC tissues compared with matched adjacent normal tissues and acts as an independent predictor of poor prognosis. The microarray data analysis and cell experiments revealed that SMC4 is closely related to the tumor cell cycle, cell adhesion, and RNA processing. These results suggests that SMC4 has important functions in both lung development and lung cancer progression. Moreover, the findings of this study demonstrate that the investigation of cancer through a comparative study between development and tumorigenesis will allow an easier identification of potential therapeutic targets and biomarkers and a more in-depth understanding of the mechanisms of carcinogenesis.

## Results

### Identification of SMC4 through construction of a co-expression network using lung development data

**Figure 1:**
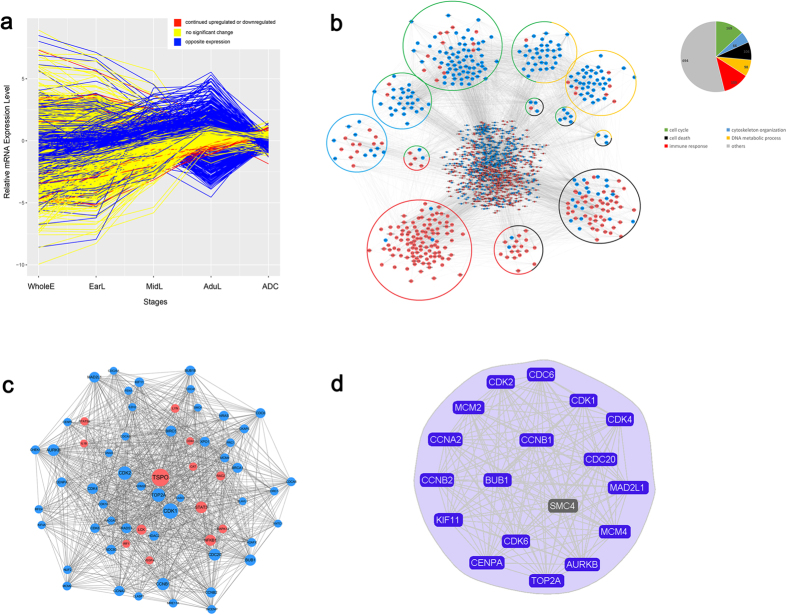
Identification of SMC4 through construction of a co-expression network using lung development data. (**a**) The co-expression network contained 1288 nodes and 8860 edges. (**b**) Scattergram of node degrees in the co-expression network. The co-expression network was scale-free. (**c**) The subnetwork contained 63 hub nodes with the top 5% of degrees. The nodes represent the genes, and the edges indicate the interactions between genes. The genes upregulated during lung development are labeled in red, whereas the downregulated genes are labeled in blue. All of the nodes are marked with node degrees. (**d**) K-clique community with 18 nodes (k = 16). These genes are closely involved in the cell cycle and chromosome organization.

By further mining the data from the 27 PTNs^23^, we focused on PTN1, PTN2, PTN25, and PTN27, in which genes were continuously upregulated or downregulated during lung development. The gene expression profiles of PTN2 and PTN25 were not significantly different between the whole embryos from postovulatory weeks (PWs) 3 to 5 (WholeE) and early fetal lung tissues at 6 to 8 PWs (EarL) stages, which may be due to the close time points used for tissue sample collection. Because tumorigenesis is considered the reverse process of embryonic development, genes that are downregulate during lung development (PTN1 and PTN2) may be upregulated in lung cancer, whereas genes that are upregulated during lung development (PTN25 and PTN27) may be downregulated in lung cancer. The statistical analysis suggested the inclusion of 1640 genes in the four PTNs; of these, 613 genes showed opposite expression patterns in lung ADCs, 31 genes were continuously upregulated or downregulated in lung ADCs, and the remaining 996 genes demonstrated no significant changes in lung ADCs compared with adult lung tissues (AduL) collected from adult patients who had undergone surgery for benign lung diseases (Fig. 1A). These results revealed that some of the genes that showed continuous change during lung development exhibited no changes in lung ADCs, which could be attributed to tumor heterogeneity and disorders of the molecular network. We could not simply ignore the potential role of the genes that presented no changes in tumorigenesis. Therefore, we used all 1640 genes that presented continuous changes during lung development for the study of lung carcinogenesis.

First, the genes were mapped to the STRING database to build the protein-protein interaction (PPI) pairs. Furthermore, we calculated Spearman’s correlation coefficient for the screened PPI pairs in 44 lung development samples to obtain a co-expression network that contained 1288 nodes and 8860 edges, which are mainly involved in the cell cycle, cytoskeleton organization, DNA metabolic process, cell death and immune system (GO: Biological process, Fig. 1B). The network exhibited a scale-free connectivity (Power  > 0.85), in accordance with the characteristics of the biological network (supplementary Fig. S1). Subsequently, we selected 63 hub nodes in the top 5% with respect to degree for the construction of a hub network (Fig. 1C) and then used CFinder to detect cliques based on the Clique Percolation Method. We then found a module with 18 nodes (Fig. 1D), including SMC4, which has the highest stringency (k = 16), was closely associated with the cell cycle and chromosome organization (GO: Biological process; supplementary Table S1). These results indicated that the SMC4 associated module play an important role in lung develpoment. Because of the intimate relationship between the developmental process and carcinogenesis, this group of genes may also participate in lung carcinogenesis. We ultimately selected SMC4, which has not yet been studied in lung cancer, for further exploration.

### SMC4 participates in multiple aspects of lung development and tumorigenesis

**Figure 2:**
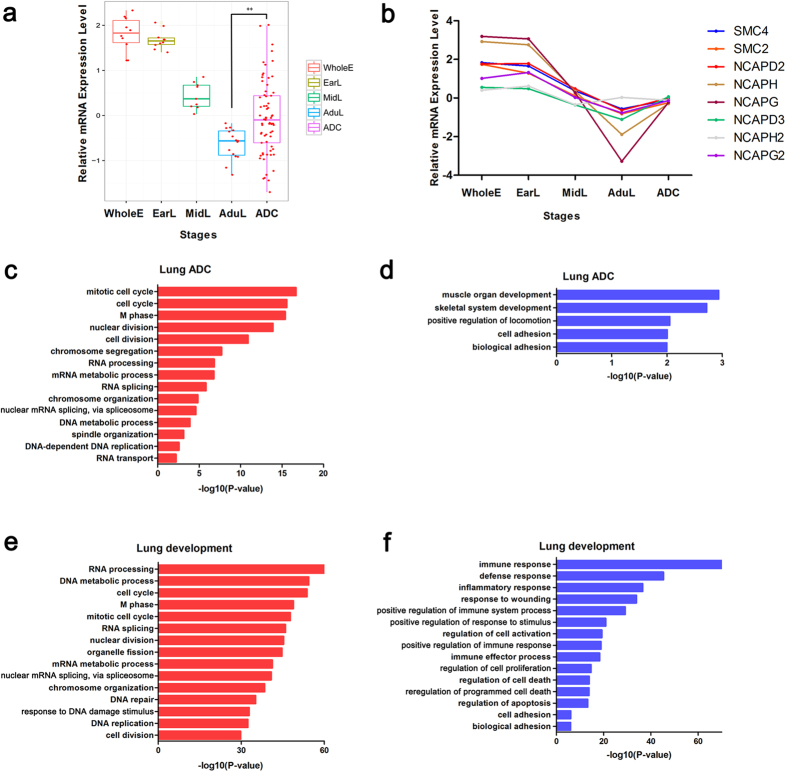
SMC4 participates in multiple aspects of lung development and tumorigenesis. (**a**) Expression levels of SMC4 during four stages of lung development and in lung ADC samples. The bars represent the minimum and maximum normalized signals. Two-tailed unpaired t-test, **p < 0.01. (**b**) Median expression level of all eight condensin subunits at four stages of lung development and in lung ADC samples. (**c,d**) Representative GO biological process (BP) terms of the genes positively correlated with SMC4. (**e,f**) Representative GO BP terms of the genes negatively correlated with SMC4. The −log10 (enrichment P-value) is displayed on the x-axis.

The microarray data showed that SMC4 and its condensin counterparts (SMC2, NCAPD2, NCAPH, NCAPG, NCAPD3, NCAPG2, and NCAPH2), with the exception of NCAPH2, belong to PTN1 and PTN2. The expression levels of SMC4 gradually decrease during lung development and are increased in lung ADCs and there was a significant difference between AduL and lung ADC (Two-tailed unpaired t-test, p = 0.0033; Fig. 2A). Other condensin subunits, with the exception of NCAPH2, show the same pattern (Fig. 2B). These results demonstrated that as a hub gene in chromosome organization and the cell cycle, SMC4 exhibited opposite expression patterns in lung tumorigenesis and development. The expression level of SMC4 is highly variable in lung ADC (Fig. 2A), which is consistent with tumor heterogeneity. We hypothesized that tumors with different SMC4 expression levels may exhibit different biological and pathological features.

To determine the relationship between SMC4 expression patterns and the biological features of cancer, the mRNA microarray data from 69 lung ADC samples were further analyzed. Spearman’s rank correlation test was performed to identify genes that were significantly correlated with SMC4. A total of 435 genes were recognized to be closely correlated with SMC4, and these included 240 positively correlated genes and 195 negatively correlated genes. Through GO analysis we found that these positively correlated genes are associated with cell cycle, chromosome segregation, RNA splicing, DNA metabolic process, spindle organization (ontology: biological process; Fig. 2C), chromosome, nuclear lumen, spindle, and spliceosome (ontology: cellular component; supplementary Fig. S2A), whereas the negatively correlated genes were associated with cell adhesion, biological adhesion (ontology: biological process; Fig. 2D) and extracellular region (ontology: cellular component; supplementary Fig. S2B). KEGG pathway analysis revealed that multiple cellular pathways, including the spliceosome, cell cycle, focal adhesion, and calcium signaling pathways, were significantly altered (supplementary Fig. S2C,D). In addition, the microarray data analysis of 44 lung development samples showed similar results (Fig. 2E,F; supplementary Fig. S2E–H). These data indicated that SMC4 participates in multiple aspects of lung development and tumorigenesis, which suggests that lung tumor cells may mimic the gene expression patterns of embryonic lung cells to enhance their competitive advantage to normal somatic cells.

### SMC4 is overexpressed in lung ADC tissue samples and acts as an independent prognostic predictor of lung ADC

**Figure 3:**
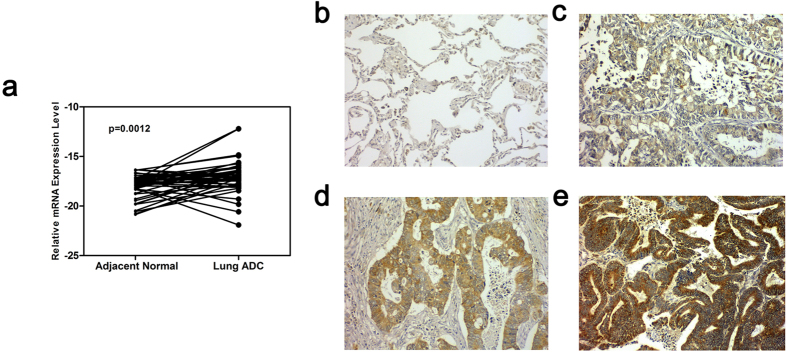
SMC4 is overexpressed in lung ADC tissues compared with adjacent normal lung tissues at both the mRNA and protein levels. (**a**) Expression levels of SMC4 mRNA in 43 lung ADC tissues and paired normal lung tissues. The expression levels were determined by real-time PCR, and the y-axis shows the −ΔCt values. 18S ribosomal RNA was used as reference gene. Two-tailed paired t-test, **p < 0.01. (**b–e**) Representative immunohistochemical staining results for SMC4 protein in (**b**) adjacent normal lung tissue with negative staining (×200), (**c**) lung ADC tissue with weak positive staining (×200), (**d**) lung ADC tissue with moderate positive staining (×200), and (**e**) lung ADC tissue with strong positive staining (×200).

To verify whether the expression levels of SMC4 differed between lung ADC samples and normal lung samples, we first performed a real-time RT-PCR assay of paired lung ADC tissues and adjacent normal lung tissues and found that the expression level of SMC4 mRNA is 1.8-fold higher in 43 lung ADC tissues compared with their adjacent normal lung tissues (Two-tailed paired t-test, p = 0.0012; Fig. 3A). 18S ribosomal RNA was used as a reference gene. A tissue microarray was used to detect the protein expression level of SMC4 by immunohistochemistry (IHC). The staining pattern was intense and clear in the cytoplasm. Negative SMC4 expression was detected in adjacent normal tissue samples, and significant SMC4 overexpression was noted in lung ADC tissue samples (Fig. 3B–E), of which 11.27% (8/71) showed strong positive staining, 57.75% (41/71) showed moderate positive staining and 30.98% (22/71) showed weak positive staining.

**Figure 4:**
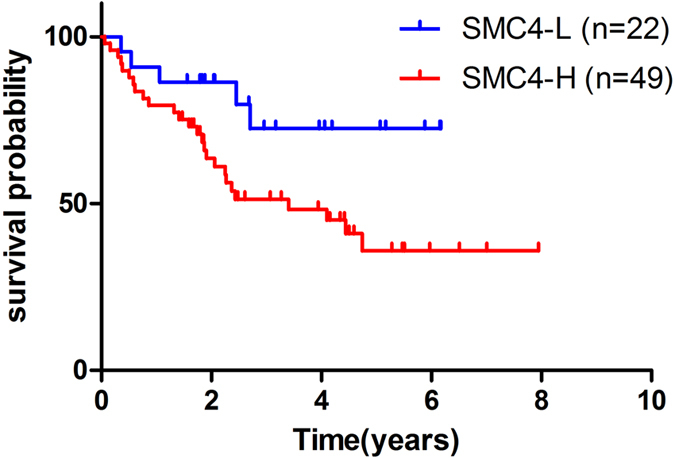
Association between SMC4 expression and lung ADC prognosis. 71 ADC patients was divided into two groups based on the immunostaining score: 0–1 points represents the SMC4-L group (n = 22, blue line), and 2–3 points represents the SMC4-H group (n = 49, red line). Higher SMC4 protein expression level is a poor prognostic factor for overall survival, as indicated by Kaplan-Meier analysis.

A further analysis showed no statistically significant associations between SMC4 expression and clinicopathological characteristics, including age, gender, TNM stage, N status, and smoking (Table 1). A statistical analysis revealed that high SMC4 expression is significantly correlated with poor survival (Fig. 4). The average survival time of the patients with low and high SMC4 expression was 59.0 and 49.8 months, respectively. Furthermore, a Cox proportional hazards model was applied to estimate the effect of SMC4 expression on survival. The hazard ratio (HR) of the SMC4 high expression (SMC4-H) group compared with the SMC4 low expression (SMC4-L) group was 2.649 (95% confidence interval [CI], 1.003–6.999, P = 0.049), indicating that high SMC4 expression increases the risk of lung ADC-related death by nearly three-fold compared with that observed with low SMC4 expression. The multivariate analysis revealed that SMC4 expression and the TNM stage are significantly associated with survival (Table 2). These findings suggest that SMC4 is an independent predictor of poor survival.

### SMC4 silencing inhibits the proliferation and invasion of lung ADC cells

**Figure 5:**
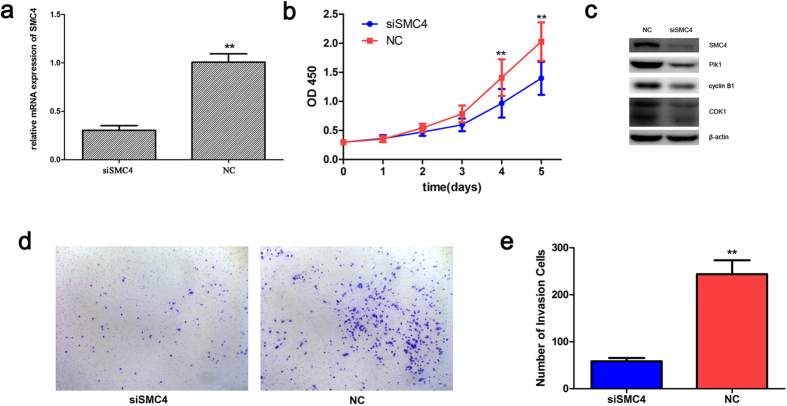
Downregulation of SMC4 inhibits the proliferation and invasion of A549 cells. The mRNA level (**a**) and protein level (**c**) of SMC4 in A549 cells were determined by real-time PCR and Western blot analysis, respectively, 48 h after the cells were transfected with siSMC4 or NC. (**b**) The CCK-8 assay was used to evaluate the proliferation of A549 cells after transfection. (**c**) Western blots showed the effect of SMC4 knockdown on mitosis-related genes in tumor cells after 48 h. β-actin was used as a control to ensure equal loading. (**d,e**) Transwell invasion assay of A549 cells 48 h after transfection with siSMC4 or NC. **P < 0.01. The data are presented as the means ± SEM from three independent experiments.

According to the analysis presented above, we hypothesized that SMC4 may be involved in the proliferation and invasion of lung ADC cells. To validate this hypothesis, A549 cells transfected with small interfering RNA (siRNA) targeting SMC4 (siSMC4) or non-targeting control (NC) were used in Cell Counting Kit-8 (CCK-8) and Transwell assays for the measurement of cell proliferation and invasion, respectively. Real-time reverse transcription-polymerase chain reaction (RT-PCR) and Western blotting analyses revealed that the treatment of A549 cells with siSMC4 resulted in a significant decrease in SMC4 mRNA and protein expression compared with the NC group cells (Fig. 5A,C). CCK-8 assays showed that the proliferation of A549 cells was significantly inhibited 72, 96, and 120 h after SMC4 knockdown compared with the proliferation of the cells in the NC group (Fig. 5B). To determine how SMC4 contributes to cell proliferation, we further examined the possible effects of SMC4 knockdown on the expression of several mitosis-related proteins. Western blot analysis revealed that the expression of Plk1 and its downstream substrates cyclinB1 and CDK1 was decreased (Fig. 5C). These results suggested that the knockdown of SMC4 blocked M phase entry and obstructed M phase progression. Moreover, the Transwell assay results revealed that the downregulation of SMC4 resulted in a significant decrease in the invasive capabilities of A549 cells (Fig. 5D,E).

### SMC4 interacts with a pre-mRNA splicing component DDX46

**Figure 6:**
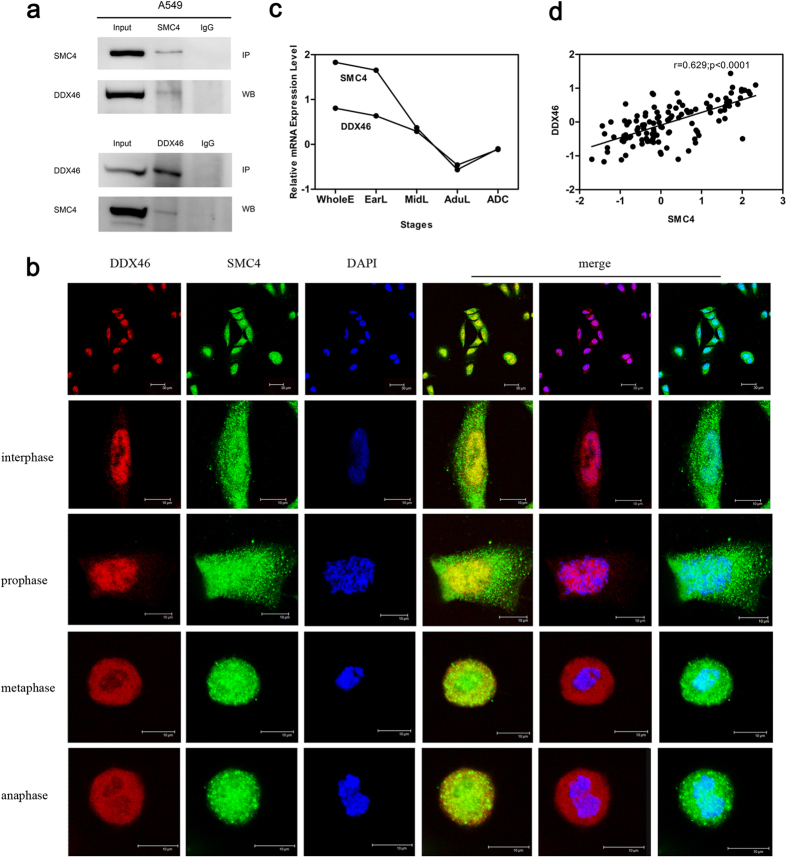
SMC4-DDX46 protein interactions *in vivo*. (**a**) Nuclear extracts of A459 cells were immunoprecipitated with anti-SMC4 or anti-DDX46 antibodies or control Ig. Co-IP samples were analyzed by Western blotting with the indicated antibodies. (**b**) Double immunofluorescence staining for endogenous SMC4 (green) and DDX46 (red) in A549 cells. Merged images are also shown. (**c**) mRNA expression levels of SMC4 and DDX46 during four stages of lung development and in lung ADC samples. The bars represent the median normalized signals. (**d**) Linear relationship of SMC4 and DDX46 in lung development samples and lung ADC samples at the mRNA expression levels, as determined by Spearman’s correlation coefficient test.

To further investigate the role of SMC4 in cancer progression, we performed co-immunoprecipitation (Co-IP) coupled mass spectrum (MS) studies in A549 cells to identify proteins that potentially interact with SMC4. To validate the MS results, we conducted reciprocal Co-IPs in A549 cells. Nuclear extracts were immunoprecipitated with normal rabbit IgG or with anti-SMC4 or anti-DDX46 antibodies. The Western blot results showed that SMC4 interacts with DDX46 (Fig. 6A). DDX46 is a component of the 17S U2 snRNP complex and plays an important role in pre-mRNA splicing before or during prespliceosome assembly^24^,^25^,^26^. In addition, red immunostaining for DDX46 and green immunostaining for SMC4 produced strong yellow staining when viewed with a double filter, indicating the co-localization of DDX46 with SMC4 in A549 cells (Fig. 6B). The microarray data analysis of 44 lung development samples and 69 lung ADC samples showed that the mRNA expression pattern of DDX46 was similar to that of SMC4 (Fig. 6C). Moreover, Spearman’s correlation coefficient showed that SMC4 is positively correlated with DDX46 at the mRNA levels (Fig. 6D). The analysis of microarray data showed that SMC4 was closely related to RNA processing; besides, we found that SMC4 interacted with a pre-mRNA splicing component DDX46. However, whether SMC4 participates in RNA processing needs to be further studied.

## Discussion

The understanding of the relationship between developmental processes and tumorigenesis can be traced back to 1858, when the concept was first proposed by Rudolf Virchow. In recent decades, growing numbers of studies have illustrated that various signaling, transcriptional, and metabolic pathways are shared between organogenesis and malignant tumors. Tumors can be regarded as special “organs” that undergo aberrant and poorly regulated organogenesis^7^,^8^,^9^,^10^,^11^,^12^,^13^,^14^. Cancer is a complex molecular network disease, and the high heterogeneity and disorder of tumor cells have caused great challenges in cancer research. Moreover, it is difficult to study the dynamic progression of tumors, particularly during the precancerous stages. Distinct from carcinogenesis, embryogenesis is a tightly regulated process. The molecular regulation network in embryos is highly ordered, whereas the heterogeneity of embryo tissue samples is low, which makes the high-throughput platform easier and more effective for exploring the key molecular network. In addition, embryo development is strictly controlled by time, and each stage is definite; thus, it is easier to study the whole embryo development process than that of a tumor. Therefore, the re-examination of distinctive processes involved in normal development might help elucidate the intrinsic features of cancer that are significantly obscured by its heterogeneity and disorder and might eventually lead to marked progress in the prevention and therapy of cancer.

Previous studies showed that cancer cells mimic the gene expression patterns of early developmental stages of the corresponding organ^10^,^11^,^12^,^13^,^14^. In our study, we hypothesized that genes that present constant changes in expression during embryonic development may play an important role in the development of precancerous lesions and cancer progression. Therefore, a further microarray data analysis of the 44 human lung developmental tissues at four different stages focused on 1640 genes that were continuously upregulated or downregulated during lung development (Fig. 1A). We found that those genes were not independent of each other and revealed many mutual regulations among them. In addition, during development, the fetal lung exhibited downregulation of the cell cycle, cytoskeleton organization, and DNA metabolic process and upregulation in the immune response and cell death (Fig. 1B), which is opposite to the findings observed during carcinogenesis^27^,^28^,^29^,^30^,^31^. This result provides evidence that tumorigenesis is the inverse process of embryo development. Furthermore, from the hub development network (Fig. 1C), we detected an densely interconnected 18-node module associated with the cell cycle and chromosome organization (Fig. 1D), and this module included SMC4. These results indicated that as a hub gene in the molecular network of lung development, SMC4 may play an important role in lung carcinogenesis.

SMC4 is a member of the SMC family. Its function and structure are highly conserved from bacteria to humans. As a chromosomal ATPase with two highly conserved nucleotide-binding Walker A and B motifs on the N-terminal and C-terminal domains, respectively, the foremost role of SMC4, together with other condensin subunits, is to convert interphase chromatin into mitotic-like condensed chromosomes and participate in mitotic sister chromatid segregation^27^,^28^. Increasing numbers of studies suggest that SMC4 also plays important roles in the non-mitotic stages of the cell cycle, such as maintenance of the silenced state of gene expression, heterochromatin organization, and DNA repair^29^,^30^,^31^,^32^. Recent studies showed abnormal expression of SMC4 in liver and colon cancers^19^,^20^,^21^,^22^, but these results do not explain the underlying molecular mechanisms of SMC4 in cancer. Thus, the role of SMC4 in carcinogenesis needs to be further explored.

In our study, we found that the expression patterns of SMC4 was gradually decreased during lung development and increased in lung ADC (Fig. 2A). The correlation analysis of 69 lung ADC samples showed that SMC4 were associated with cell cycle progression, RNA splicing, DNA metabolic process, cell adhesion and extracellular matrix. Moreover, the microarray data analysis of 44 lung development samples showed similar results (Fig. 2C–F; Fig. S2). These results show that SMC4 may play a similar role in lung development and lung tumorigenesis, suggesting that lung tumor cells mimic the gene expression patterns of embryonic lung cells to enhance their competitive advantage to normal somatic cells. Therefore, lung ADC patients with different SMC4 expression levels may exhibit different pathological features and survival outcomes.

Previous studies showed that SMC4 is involved in tumorigenesis, and the overexpression of SMC4 has been detected in hepatocellular carcinoma (HCC) and colorectal cancer^19^,^22^. Our study showed that SMC4 is significantly increased in lung ADC compared with matched adjacent normal lung tissues at both the mRNA and protein levels (Fig. 3). Moreover, a high SMC4 protein level in lung ADC patients was identified as an independent predictor of poor prognosis (Fig. 4; Table 2). siRNA knockdown experiments showed that SMC4 inhibition can suppress the proliferation of A549 cells by downregulating the mitosis-related protein Plk1 and its downstream substrates cyclin B1 and CDK1 (Fig. 5B,C), and suppress the invasion of A549 cells (Fig. 5D,E). These results were consistent with previous reports that SMC4 knockdown was able to reduce HCC and colorectal cancer cell viability^19^,^21^. In our study, we first found a connection between SMC4 and lung cancer, but the underlying specific molecular mechanism and the potential use of SMC4 as a prognostic marker in lung ADC require further investigation.

In conclusion, we reveal that SMC4, which is essentially involved in lung development, is closely associated with lung ADC progression and prognosis. Our study suggests an intimate relationship between the developmental process and carcinogenesis. Genes play important roles in development; for example, SMC4 may also participate in tumorigenesis. The factor that causes tumor cells to mimic the gene expression patterns obtained in the corresponding organ during the early developmental stages and then exhibit embryonic tissue organization remains unclear. Therefore, the identification of genes that are essentially involved in development through a comparative study between development and cancer would be a practical strategy for discovering potential therapeutic targets and biomarkers and obtaining a better understanding of the mechanisms of carcinogenesis.

## Methods

### Patients and specimens

Pairs of lung ADC tissues and adjacent normal lung tissues (at least 5 cm from the tumor) were obtained from 43 patients. All of the tissues were obtained from patients diagnosed with lung ADC who underwent surgery at the Cancer Institute and Hospital, Chinese Academy of Medical Sciences, between 2004 and 2009. No patients received chemotherapy or radiotherapy prior to surgery. The surgically resected tissue samples were snap-frozen in liquid nitrogen immediately after surgery and stored at −70 °C. A fraction of the tissues was subjected to pathological examination by two independent and experienced pathologists blinded to the study conditions. Samples meeting the diagnostic criteria for neoplasia (neoplastic cells > 70%) were included. This study was reviewed and approved by the Ethics Committee of the Cancer Institute and Hospital, Chinese Academy of Medical Sciences, and all of the patients provided written informed consent. All the methods were carried out in accordance with the approved guidelines.

### RNA isolation and RT-PCR

Total RNA was extracted and reverse transcribed. Then the real-time PCR was conducted as described in our previous report^33^. Melting curves were generated, and the relative target gene mRNA level was normalized to that of 18S ribosomal RNA. All data are presented as the average of three replicates. The gene-specific primers used for the amplifications were the following:

SMC4-F, 5′-CAGAACGGCCTGCAGAGATA-3′;

SMC4-R, 5′-TCAAGCATACCCTCATCGTGT-3′;

18S-F, 5′-CTGAGAAACGGCTACCACATCC-3′;

18S-R, 5′-GCACCAGACTTGCCCTCCA-3′.

### Immunohistochemistry and scoring

Paraffin-embedded lung ADC tissue microarray (TMA) was purchased from Shanghai Super BioTek (Shanghai, China). This TMA was commercially preconstructed by dot-arrayed tissues in parallel from 75 cases of lung ADC patients for whom there were clinicopathological data and detailed follow-up information. Because tissue dropping was found in four cases, we ultimately analyzed 71 cases of arrayed tissues from lung ADC patients. More detailed information on the arrayed tumor tissues and paired tumor-adjacent normal tissues is provided in the results section.

After deparaffinization and rehydrationin in xylene and ethanol, the TMA sections were incubated with 3% H_2_O_2_ to block endogenous peroxidase activity followed by heat-induced antigen retrieval. After cooling to room temperature, the sections were treated with 5% normal goat serum and incubated with a primary antibody for SMC4 at a 1:25 dilution (Abcam, UK) overnight at 4 °C. A horseradish peroxidase-conjugated secondary antibody was then applied, and the chromogenic reaction was performed using a commercial diaminobenzidine (DAB) kit (Zhongshan Golden Bridge Biotechnology Company, Beijing, China). The sections were then counterstained with hematoxylin.

The IHC-stained sections were analyzed and scored independently by two experienced pathologists without prior knowledge of the patients’ clinicopathological data. The intensity of the immunostaining was scored as follows: 0 (“−”), 1 (“+”), 2 (“++”), and 3 (“+++”). Final pathological scores of at least 2 were regarded as representing “high expression”. The percentage of positive cells was not calculated because all of the samples showed areas of homogeneous staining.

### Cell culture and siRNA knockdown

The human lung cancer cell line A549 was obtained from the China Infrastructure of Cell Line Resource (Beijing, China). The cells were cultured in RPMI-1640 supplemented with 10% fetal bovine serum (Life Technologies, Grand Island, NY, USA). For the knockdown experiments, the cells were seeded in six-well plates and transfected with siRNA using the Lipofectamine RNAiMAX transfection reagent (Invitrogen, Carlsbad, CA, USA) according to the manufacturer’s instructions.

### CCK-8 assay

The proliferation ability of A549 cells was measured using the CCK-8 (Dojindo, Kumamoto, Japan). Briefly, A549 cells were seeded on 96-well plates at a density of 2000 cells per well. After adhesion for 24 h, the cells were transfected with siSMC4 or NC. Following incubation for 0 to 120 h at intervals of 24 h, the cells were stained with 10 μl of CCK-8 solution in 90 μl of culture medium for 2.5 h at 37 °C. Cell proliferation was measured based on the absorbance at 450 nm using a microplate reader (BioTek, Synergy HT, VT, USA).

### *In vitro* invasive assay

Corning BioCoat Matrigel Invasion Chambers (24 wells, 8.0-μm pore size, Corning,USA) were used for a transwell invasion assay following the manufacturer’s instructions. Briefly, 48 h after transfection, 5 × 10^4^ A549 cells suspended in 500 μl of serum-free RPMI-1640 medium were seeded into the upper chambers, whereas 750 μl of complete medium with 10% FBS was injected into the lower chambers as a chemoattractant. After incubation for 22 h, the cells that had invaded through the membrane were fixed with cold methanol and stained with 0.1% crystal violet, and the cells in no less than five fields under a light microscope were counted.

### Western blotting

The total protein from the A549 cells was extracted using RIPA lysis buffer (Applygen, Beijing, China) and quantified using the Pierce BCA Protein Assay Kit (Thermo Fisher Scientific, USA). Subsequently, equal amounts of the proteins were separated via sodium dodecyl sulfate-polyacrylamide gel electrophoresis (SDS-PAGE) and transferred onto a 0.25-μm polyvinylidene difluoride (PVDF) membrane (Millipore, Bedford, MA, USA) through a wet trans-blot system (BioRad, USA). The membranes were then blocked, incubated with antibodies against SMC4 (Abcam, UK), Plk1 (Abcam, UK), cyclin B1 (Abcam, UK) and CDK1 (Abcam, UK) overnight at 4 °C and then with an HRP-conjugated secondary antibody (Zhongshan Golden Bridge Biotechnology Company, Beijing, China) and analyzed using an enhanced chemiluminescence (ECL) detection kit (Applygen, Beijing, China). An image was finally obtained with an ImageQuant LAS 4000mini system (GE Healthcare, Piscataway, NJ, USA). β-actin (Santa Cruz, CA, USA) was used as an internal control.

### Co-immunoprecipitation

To obtain nuclear extracts, A549 cells were lysed in NE-PER Nuclear and Cytoplasmic Extraction Reagents (Thermo Fisher Scientific, USA) according to the manufacturer’s protocol. The nuclear extracts were pre-cleared with Protein A/G PLUS Agarose (Santa Cruz, CA, USA), and immune complexes were obtained by incubation with anti-SMC4 antibody (Abcam, UK) and pulled down by overnight incubation at 4 °C with Protein A /G PLUS Agarose. Normal rabbit IgG was used as a negative control. Subsequently, the immunoprecipitates were washed with 1xTBS buffer (Thermo Fisher Scientific, USA), mixed with 2x Laemmli Sample Buffer (BioRad, USA), boiled for 10 min, and separated by SDS-PAGE. Primary antibodies against SMC4 (Abcam, UK) and DDX46 (Novus Biologicals, USA) were used for Western blotting. Reciprocal Co-IPs were performed in the same manner.

### Double immunofluorescence staining

A549 cells were washed in PBS and fixed in 4% buffered paraformaldehyde at 37 °C, then permeabilized with 0.5% TritonX-100. The cells were blocked with 5% goat serum in PBS for 1 h and incubated with anti-SMC4 (Abcam, UK) and anti-DDX46 (Santa Cruz, CA, USA) antibodies overnight at 4 °C. Following five washes in PBS, the cells were incubated with Alexa 488-conjugated goat anti-rabbit IgG (for SMC4) and Alexa 594-conjugated goat anti-mouse IgG (for DDX46) (Zhongshan Golden Bridge Biotechnology Company, Beijing, China) for 1 h at 37 °C. After washing, the slides were immediately mounted in mounting medium with DAPI (Zhongshan Golden Bridge Biotechnology Company, Beijing, China). The cells were examined using a confocal laser-scanning microscope (LEICA TCS SP2, Germany), and images were captured using Leica Confocal Software.

### Analysis of the public microarray dataset

The human lung samples included 44 developing lung tissues at four different stages and 69 lung ADC tissues. The developing samples included WholeE, EarL, middle fetal lung tissues at 16 to 24 PWs (MidL), and AduL. We obtained raw GSE43767 data from the Gene Expression Omnibus (GEO) database (http://www.ncbi.nlm.nih.gov/geo/). mRNA expression data normalization and quality control were performed using GeneSpring’s default settings.

The STRING v10 database was used to screen protein-protein interaction (PPI) pairs, and the cutoff criteria were text mining >200 and combined score >400 (http://string.embl.de/). Spearman’s rank correlation test was applied to filter PPI pairs and seek SMC4-correlated genes, and a correlation was considered statistically significant if the false-discovery rate (FDR) adjusted p value was less than 0.05 (R project). A hub network was obtained and visualized using Cytoscape 3.2.1. Network cluster detection was performed based on CFinder 2.0.6, and DAVID was applied to analyze the Gene Ontology (GO) enrichment of gene functions and KEGG pathways (https://david.ncifcrf.gov/).

### Statistical analysis

Statistical analyses were performed using SPSS version 17.0 software (SPSS, Chicago, IL, USA). The differences between lung ADC tissues and AduL were assessed through a Two-tailed unpaired t-test. The differences between lung ADC tissues and matched normal lung tissues were assessed through a Two-tailed paired t-test. To assess the association of clinicopathological data with SMC4 expression, the Chi-square test was used for categorical variables, and the Mann-Whitney U test was used for continuous variables. Survival curves were obtained by the Kaplan-Meier method, and the differences in survival were analyzed by the log-rank test. Multivariate analyses were conducted using a Cox proportional hazards model. Differences were considered statistically significant if the p value was less than 0.05.

## Additional Information

**How to cite this article**: Zhang, C. *et al*. SMC4, which is essentially involved in lung development, is associated with lung adenocarcinoma progression. *Sci. Rep.* **6**, 34508; doi: 10.1038/srep34508 (2016).

## Supplementary Material

Supplementary Information

## Figures and Tables

**Table 1:** Relationship between SMC4 expression and clinicopathological characteristics of 71 lung ADC patients.

Characteristics	SMC4-H (No. of Cases (%))	SMC4-L (No. of Cases (%))	P-value
	n = 49	n = 22	
Age			0.96
Mean ± SD (years)	57.88 ± 10.07	58.82 + 7.77	
Gender			0.41
Female	26(53.06%)	14(63.64%)	
Male	23(46.94%)	8(36.36%)	
TNM Stage			0.23
Stage I	26(53.06%)	15(68.18%)	
Stage II+III+IV	23(46.94%)	7(31.82%)	
N Status			0.27
N0	29(59.18%)	16(72.73%)	
N1	20(40.82%)	6(27.27%)	
Smoking			0.71
Yes	20(40.82%)	10(45.45%)	
No	29(59.18%)	12(54.55%)	

**Table 2:** Multivariate Cox regression proportional hazards analysis of 71 lung ADC patients.

Characteristics	P-value	HR	95.0% CI
SMC4	0.049	2.649	1.003–6.999
Age	0.853	1.004	0.964–1.046
Gender	0.805	1.127	0.437–2.905
TNM Stage	0.041	0.28	0.082–0.951
N Status	0.158	2.378	0.714–7.921
Smoking	0.988	0.992	0.363–2.713

## References

[b1] Sardenberg R. A., Mello E. S. & Younes R. N. The lung adenocarcinoma guidelines: what to be considered by surgeons. Journal of thoracic disease 6, S561–567, doi: 10.3978/j.issn.2072-1439.2014.08.25 (2014). 25349707 PMC4209382

[b2] Torre L. A. . Global cancer statistics, 2012. CA: a cancer journal for clinicians 65, 87–108, doi: 10.3322/caac.21262 (2015). 25651787

[b3] Galvan A. . Gene expression signature of non-involved lung tissue associated with survival in lung adenocarcinoma patients. Carcinogenesis 34, 2767–2773, doi: 10.1093/carcin/bgt294 (2013). 23978379

[b4] Goldstraw P. . Non-small-cell lung cancer. Lancet (London, England) 378, 1727–1740, doi: 10.1016/s0140-6736(10)62101-0 (2011). 21565398

[b5] Marin J. J., Briz O., Monte M. J., Blazquez A. G. & Macias R. I. Genetic variants in genes involved in mechanisms of chemoresistance to anticancer drugs. Current cancer drug targets 12, 402–438 (2012). 22229248 10.2174/156800912800190875

[b6] Tanaka K., Kumano K. & Ueno H. Intracellular signals of lung cancer cells as possible therapeutic targets. Cancer science 106, 489–496, doi: 10.1111/cas.12643 (2015). 25707772 PMC4452148

[b7] Naxerova K. . Analysis of gene expression in a developmental context emphasizes distinct biological leitmotifs in human cancers. Genome biology 9, R108, doi: 10.1186/gb-2008-9-7-r108 (2008). 18611264 PMC2530866

[b8] Hartwell K. A. . The Spemann organizer gene, Goosecoid, promotes tumor metastasis. Proceedings of the National Academy of Sciences of the United States of America 103, 18969–18974, doi: 10.1073/pnas.0608636103 (2006). 17142318 PMC1748161

[b9] Liu S. . Hedgehog signaling and Bmi-1 regulate self-renewal of normal and malignant human mammary stem cells. Cancer research 66, 6063–6071, doi: 10.1158/0008-5472.can-06-0054 (2006). 16778178 PMC4386278

[b10] Borczuk A. C. . Non-small-cell lung cancer molecular signatures recapitulate lung developmental pathways. The American journal of pathology 163, 1949–1960, doi: 10.1016/s0002-9440(10)63553-5 (2003). 14578194 PMC1892411

[b11] Hu M. & Shivdasani R. A. Overlapping gene expression in fetal mouse intestine development and human colorectal cancer. Cancer research 65, 8715–8722, doi: 10.1158/0008-5472.can-05-0700 (2005). 16204040

[b12] Kho A. T. . Conserved mechanisms across development and tumorigenesis revealed by a mouse development perspective of human cancers. Genes & development 18, 629–640, doi: 10.1101/gad.1182504 (2004). 15075291 PMC387239

[b13] Liu H., Kho A. T., Kohane I. S. & Sun Y. Predicting survival within the lung cancer histopathological hierarchy using a multi-scale genomic model of development. PLoS medicine 3, e232, doi: 10.1371/journal.pmed.0030232 (2006). 16800721 PMC1483910

[b14] Monzo M. . Overlapping expression of microRNAs in human embryonic colon and colorectal cancer. Cell research 18, 823–833, doi: 10.1038/cr.2008.81 (2008). 18607389

[b15] Hirano M., Anderson D. E., Erickson H. P. & Hirano T. Bimodal activation of SMC ATPase by intra- and inter-molecular interactions. The EMBO journal 20, 3238–3250, doi: 10.1093/emboj/20.12.3238 (2001). 11406600 PMC150201

[b16] Lowe J., Cordell S. C. & van den Ent F. Crystal structure of the SMC head domain: an ABC ATPase with 900 residues antiparallel coiled-coil inserted. Journal of molecular biology 306, 25–35, doi: 10.1006/jmbi.2000.4379 (2001). 11178891

[b17] Steffensen S. . A role for Drosophila SMC4 in the resolution of sister chromatids in mitosis. Current biology: CB 11, 295–307 (2001). 11267866 10.1016/s0960-9822(01)00096-3

[b18] Hirano T. Condensins: universal organizers of chromosomes with diverse functions. Genes & development 26, 1659–1678, doi: 10.1101/gad.194746.112 (2012). 22855829 PMC3418584

[b19] Feng X. D. . Structural maintenance of chromosomes 4 is a predictor of survival and a novel therapeutic target in colorectal cancer. Asian Pacific journal of cancer prevention: APJCP 15, 9459–9465 (2014). 25422241 10.7314/apjcp.2014.15.21.9459

[b20] Jinushi T. . Low expression levels of microRNA-124-5p correlated with poor prognosis in colorectal cancer via targeting of SMC4. Cancer medicine 3, 1544–1552, doi: 10.1002/cam4.309 (2014). 25081869 PMC4298381

[b21] Zhou B. . A novel miR-219-SMC4-JAK2/Stat3 regulatory pathway in human hepatocellular carcinoma. Journal of experimental & clinical cancer research: CR 33, 55, doi: 10.1186/1756-9966-33-55 (2014). 24980149 PMC4096530

[b22] Zhou B. . Overexpression of the structural maintenance of chromosome 4 protein is associated with tumor de-differentiation, advanced stage and vascular invasion of primary liver cancer. Oncology reports 28, 1263–1268, doi: 10.3892/or.2012.1929 (2012). 22842912

[b23] Feng L. . Gene expression profiling in human lung development: an abundant resource for lung adenocarcinoma prognosis. PloS one 9, e105639, doi: 10.1371/journal.pone.0105639 (2014). 25141350 PMC4139381

[b24] Dalbadie-McFarland G. & Abelson J. PRP5: a helicase-like protein required for mRNA splicing in yeast. Proceedings of the National Academy of Sciences of the United States of America 87, 4236–4240 (1990). 2349233 10.1073/pnas.87.11.4236 PMC54083

[b25] Will C. L. . Characterization of novel SF3b and 17S U2 snRNP proteins, including a human Prp5p homologue and an SF3b DEAD-box protein. The EMBO journal 21, 4978–4988 (2002). 12234937 10.1093/emboj/cdf480 PMC126279

[b26] Xu Y. Z. . Prp5 bridges U1 and U2 snRNPs and enables stable U2 snRNP association with intron RNA. The EMBO journal 23, 376–385, doi: 10.1038/sj.emboj.7600050 (2004). 14713954 PMC1271757

[b27] Adams J. M. & Cory S. The Bcl-2 apoptotic switch in cancer development and therapy. Oncogene 26, 1324–1337, doi: 10.1038/sj.onc.1210220 (2007). 17322918 PMC2930981

[b28] Bindea G., Mlecnik B., Fridman W. H., Pages F. & Galon J. Natural immunity to cancer in humans. Current opinion in immunology 22, 215–222, doi: 10.1016/j.coi.2010.02.006 (2010). 20207124

[b29] Chiotaki R., Polioudaki H. & Theodoropoulos P. A. Differential nuclear shape dynamics of invasive andnon-invasive breast cancer cells are associated with actin cytoskeleton organization and stability. Biochemistry and cell biology = Biochimie et biologie cellulaire 92, 287–295, doi: 10.1139/bcb-2013-0120 (2014). 25053513

[b30] Hanahan D. & Weinberg R. A. Hallmarks of cancer: the next generation. Cell 144, 646–674, doi: 10.1016/j.cell.2011.02.013 (2011). 21376230

[b31] Mokady D. & Meiri D. RhoGTPases - A novel link between cytoskeleton organization and cisplatin resistance. Drug resistance updates: reviews and commentaries in antimicrobial and anticancer chemotherapy 19, 22–32, doi: 10.1016/j.drup.2015.01.001 (2015). 25660168

[b32] Meyer B. J. Sex in the wormcounting and compensating X-chromosome dose. Trends in genetics: TIG 16, 247–253 (2000). 10827451 10.1016/s0168-9525(00)02004-7

[b33] Li L. . DLK1 promotes lung cancer cell invasion through upregulation of MMP9 expression depending on Notch signaling. PloS one 9, e91509, doi: 10.1371/journal.pone.0091509 (2014). 24621612 PMC3951400

