# Supplementary material for: SMC4, which is essentially involved in lung development, is associated with lung adenocarcinoma progression
Source: Sci Rep. 2016 Sep 30;6:34508. doi: 10.1038/srep34508 (PMC5043270; doi:10.1038/srep34508)
Supplement: Supplementary Information [file srep34508-s1.pdf]

***Title*:** SMC4, which is essentially involved in lung development, is associated with lung adenocarcinoma progression

***Authors*:** Chengli Zhang[#], Manchao Kuang[#], Meng Li, Lin Feng[*], Kaitai Zhang[*] & Shujun Cheng[*]

Supplementary Figure S1. Scattergram of node degrees in the co-expression network. The co-expression network was scale-free.

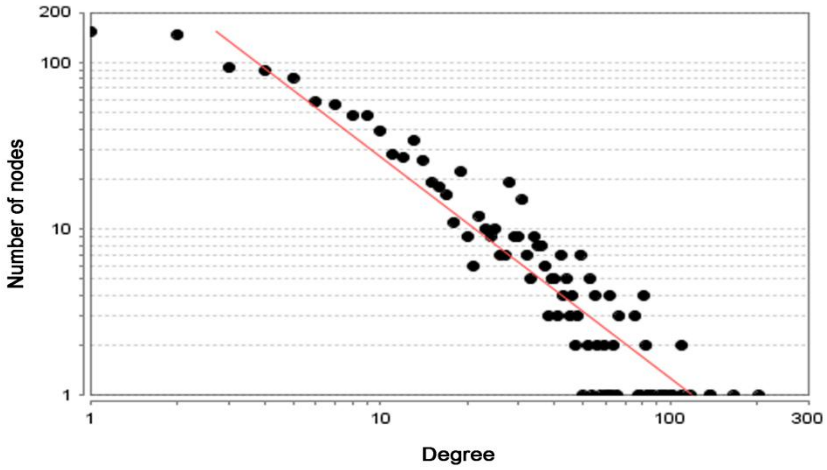

Supplementary Figure S2. SMC4 participates in multiple aspects of lung development and tumorigenesis. (a-d) Representative GO CC and KEGG terms of the genes significantly correlated with SMC4 in lung ADC. (e-h) Representative GO CC and KEGG terms of the genes significantly correlated with SMC4 in lung development. Positively correlated terms were labeled in red, while negatively correlated terms were labeled in blue.

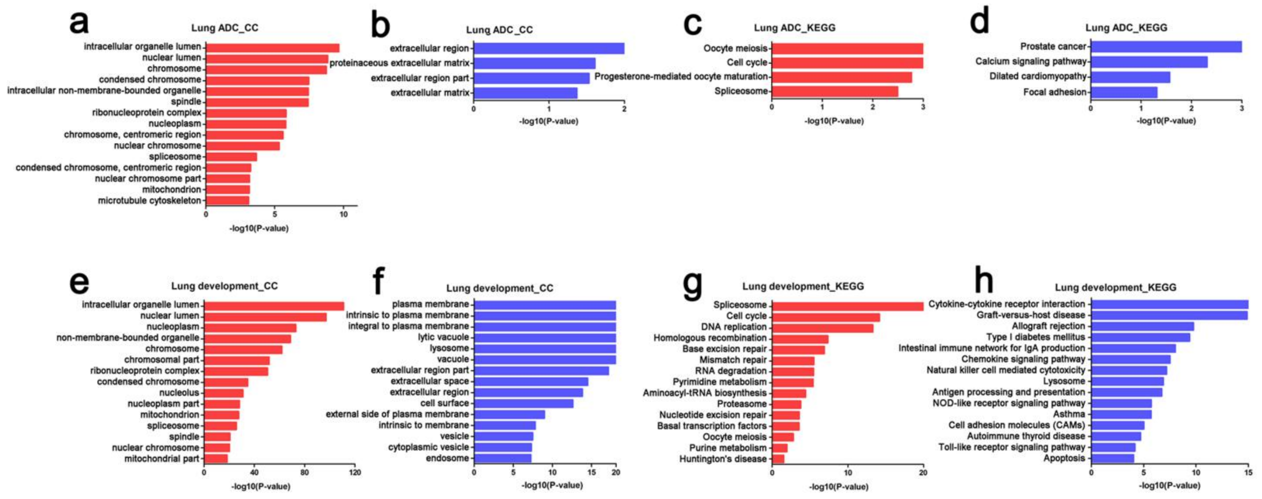

Supplementary Table.S1

GO enrichment analysis of the 18 genes in the SMC4 associated module according to biological process

GO accession	GO term	FDR
GO:0000278	Mitotic cell cycle	2.52E-16
GO:0051301	Cell division	1.57E-15
GO:0007049	Cell cycle	1.44E-13
GO:0007067	Mitosis	5.88E-13
GO:0000280	Nuclear division	5.88E-13
GO:0000075	Cell cycle checkpoint	0.0071
GO:0006260	DNA replication	0.1273
GO:0006323	DNA packaging	0.5198
GO:0031570	DNA integrity checkpoint	2.7985
GO:0051276	Chromosome organization	4.0789
